# Calcium Imaging of Nerve-Mast Cell Signaling in the Human Intestine

**DOI:** 10.3389/fphys.2017.00971

**Published:** 2017-11-29

**Authors:** Sabine Buhner, Natasja Barki, Wolfgang Greiter, Pieter Giesbertz, Ihsan E. Demir, Güralp O. Ceyhan, Florian Zeller, Hannelore Daniel, Michael Schemann

**Affiliations:** ^1^Human Biology, Technische Universität München, Freising, Germany; ^2^Molecular Nutrition Unit, Technische Universität München, Freising, Germany; ^3^Department of General Surgery, University Hospital Rechts der Isar, Technische Universität München, Munich, Germany; ^4^Surgery, Academic Hospital Freising, Freising, Germany

**Keywords:** enteric nervous system, neuropeptides, Ca^2+^ imaging, neuro-immune, CGRP, SP, VIP, human gut

## Abstract

**Introduction:** It is suggested that an altered microenvironment in the gut wall alters communication along a mast cell nerve axis. We aimed to record for the first time signaling between mast cells and neurons in intact human submucous preparations.

**Methods:** We used the Ca^2+^ sensitive dye Fluo-4 AM to simultaneously image changes in intracellular calcium [Ca^+2^]_i_ (%ΔF/F) in neurons and mast cells. Data are presented as median with interquartile ranges (25/75%).

**Results:** We recorded nerve responses in 29 samples upon selective activation of 223 mast cells by IgE receptor cross linking with the antibody mAb22E7. Mast cells responded to mAb22E7 with a median [Ca^+2^]_i_ increase of 20% (11/39) peaking 90 s (64/144) after the application. Only very few neurons responded and the median percentage of responding neuronal area was 0% (0/5.9). Mast cell activation remained in the presence of the fast sodium channel blocker tetrodotoxin. Specific neuronal activation by transmural electrical field stimulation (EFS) in 34 samples evoked instantaneously [Ca^+2^]_i_ signals in submucous neurons. This was followed by a [Ca^+2^]_i_ peak response of 8%ΔF/F (4/15) in 33% of 168 mast cells in the field of view. The mast cell response was abolished by the nerve blocker tetrododoxin, reduced by the Calcitonin Gene-Related Peptide receptor 1 antagonist BIBN-4096 and the Vasoactive Intestinal Peptide receptor antagonist PG97-269, but not by blockade of the neurokinin receptors 1–3.

**Conclusion:** The findings revealed bidirectional signaling between mast cells and submucous neurons in human gut. In our macroscopically normal preparations a nerve to mast cell signaling was very prominent whereas a mast cell to nerve signaling was rather rare.

## Introduction

The gut has two unique features. It is the largest immune competent organ in our body and it harvests an enormous number of nerves. Most of the nerve structures belong to the enteric nervous system (ENS) which regulates gut functions autonomously. The remaining extrinsic nerves connect the central nervous system with the gut and belong either to the afferent gut-brain or the efferent brain-gut axis. Both the sensory as well as the motor pathways ramify extensively once entering the gut wall. The ENS consists of two ganglionated plexus: the myenteric plexus between the two outer muscle layers and the submucous plexus close to the mucosa. The enteric immune system is an effective defense system maintaining a functional barrier and thus protecting the host from harmful invaders and noxious substances. Conceptually, it is important to realize that all three systems—ENS, extrinsic nerves, and enteric immune system—enter into an intimate anatomical and functional association (Margolis et al., 2016). Over the years, mast cells (MCs), have received a lot of attention as an important player for neuroimmune interactions in the gut. They are located primarily in perivascular spaces, ~2–3% of all cells in the lamina propria and about 1% of all cells in the submucosa (Bischoff et al., 1996) are MCs. Deeper layers almost lack MCs under normal conditions (Buhner and Schemann, 2012; Schemann and Camilleri, 2013). An estimated 90% of intestinal mucosal MCs are located at least within 2 μm (Stead et al., 1989; Dvorak et al., 1992).

There is strong evidence for nerve-MC interaction, particularly in diseases such as food allergy (Bischoff et al., 2000; Torrente et al., 2014), inflammatory bowel disease (IBD; Dvorak et al., 1978; Raithel et al., 2001), and irritable bowel syndrome (IBS; Pang et al., 1996; Schemann and Camilleri, 2013). Often, these conditions are accompanied by an increase in number and/or activity of MCs as for example in IBS (Barbara et al., 2004, 2007; Guilarte et al., 2007). Most of the studies on nerve-MC signaling focused on experiments in cell culture and rodent animal models (De Jonge et al., 2004; Furuno et al., 2004; Bell et al., 2015). These studies also proposed Substance P (SP) (Furuno et al., 2004) and Calcitonin Gene-Related Peptide (CGRP; De Jonge et al., 2004) as mediators for nerve to MC signaling. Animal studies supported an active role of MC to nerve signaling in colonic secretory responses to antigen application in sensitized rats (Bell et al., 2015) or mice (Perdue et al., 1991) and enhanced excitability of submucous neurons in sensitized guinea pigs (Frieling et al., 1994). One study showed in guinea pig ileum and colon a functional extrinsic nerve-MC-ENS pathway based on the finding that stimulation of extrinsic nerves caused release of MC proteases which in turn activated submucous neurons through the protease activated receptor (PAR) 1 and PAR2 (Wang et al., 2014). Stimulation of afferent nerves in human intestine released, besides SP and CGRP (Wang et al., 2014) also Vasoactive Intestinal Peptide (VIP) (Maggi et al., 1989).

It is striking that the concepts on nerve-MC interactions have never been demonstrated at a cellular level in intact tissue preparations and even more await validation in the human gut. At functional and molecular levels, extrapolation from animal models is problematic because MCs as well as enteric neurons exhibit species-specific features of release mechanisms, mediator profile, and neuropharmacology (Bischoff, 2007; Buhner and Schemann, 2012). For example in cultured human intestinal MCs exogenous application of neurotransmitters did not cause mediator release under basal conditions (Bischoff et al., 2004). However, MCs started to express neurokinin receptors after exposure to growth factors or IL4 (Bischoff et al., 2004). Thus, the plasticity of cultured MCs may have compromised expression of relevant receptors. Development, differentiation, and functional reactivity of MCs are critically dependent on the microenvironment (Bischoff, 2007). MCs are extremely plastic and adapt synthesis and release of their mediators to tissue conditions. All the above suggest that investigation of bidirectional communication between MCs and enteric neurons require recording of their activity in intact tissue where both cell types experience their physiological milieu.

We previously demonstrated that the mast cell mediators histamine and tryptase as well as a mediator cocktail release from human intestinal mast cells upon IgE receptor crosslinking by mAb22E7 activated human submucous neurons (Schemann et al., 2005; Breunig et al., 2007; Ostertag et al., 2017).

To our knowledge, direct recordings of neve-MC communication has not been performed and certainly not in intact human intestinal preparations. Therefore, the overall goal of the present project was to study nerve-MC signaling in submucous plexus preparations from human intestine. We used the submucous layer as the density of mast cells was highest in this region (Buhner and Schemann, 2012; Schemann and Camilleri, 2013). After selective stimulation of MCs or neurons we used [Ca^+2^]_i_ imaging to record subsequent cell activation. The participation and role of particular mediators involved in the signaling pathway were identified pharmacologically. We could show for the first time nerve-MC interaction on a cellular level in intact human submucous plexus.

## Methods

### Human tissue samples

All studies on nerve and MC activity were performed using surgical specimens of human colon (*n* = 46), ileum (*n* = 23), jejunum (*n* = 12), or duodenum (*n* = 2), obtained from 83 patients (39 females, 44 males; mean age 64 years (range 29–88 years) undergoing abdominal surgery at the Medical Clinic in Freising and the Medical Clinic of the Technische Universität München. Diagnoses that led to the surgery were as follows: carcinomas of large intestine (53), stomach (4), appendix (1), pancreas (10), adrenal gland (1), or fat tissue (1), diverticular disease (1), polyposis (2), stoma relocation (2), abdominal abscess (2), stenosis (2); fistula (1) and unspecified bleeding (1) and unspecified reasons (2). Samples were taken from macroscopically normal, unaffected areas as determined by visual inspection by the pathologists. The protocol was approved by the ethics committee of the Technische Universität München (project approval 5242/11). Informed written consent was obtained from all subjects, and the studies conformed to the standards set by the Declaration of Helsinki. After removal, the surgical specimens were placed in cold aerated sterile HEPES-Krebs solution containing in mM: 135 NaCl, 5.4 KCl; 1.0 Mg Cl_2_·6H_2_O, 1.2 NaH_2_PO_4_, 1.25 CaCl_2_·2H_2_O, 12.2 Glucose, and 3 HEPES (all from Sigma-Aldrich Chemie GmbH, Darmstadt, Germany) and 10 ml/l antibiotic-antimycotic mix (mg/L: 25 amphotericin B, 107 U/L penicillin G, 10,000 streptomycin in physiological saline; CCPro, Oberdorla, Germany). They were immediately transported to the laboratory for experiments. Under continuous superfusion with ice-cold carbogen-aerated Krebs solution containing in mM: 117 NaCl, 4.7 KCl, 1.2 MgCl_2_·6H_2_O, 1.2 NaH_2_PO_4_, 25 NaHCO_3_, 2.5 CaCl_2_·2H_2_O and 11 glucose (all from Sigma), the surgical specimens were microscopically dissected by removing the mucosa and the muscular layers to obtain a whole mount preparation of the inner submucosal plexus. The final preparations (10 × 20 mm) were pinned on silicone rings (Buhner et al., 2009).

### Calcium imaging of MC and neuronal activity

The fluorescent calcium indicator Fluo 4-acetoxymethyl (AM) (Invitrogen, Darmstadt, Germany) was used to monitor changes in intracellular calcium ([Ca^2+^]_i_; Michel et al., 2011). As previously described in detail (Ostertag et al., 2017) the submucous plexus preparations were incubated at room temperature in the dark with 10 μM Fluo-4 AM for 45 min and subsequently washed for 20 min. For incubation, washing, and superfusion during the experiments a carbogen-aerated Krebs buffer (117 NaCl, 4.7 KCl, 1.2 MgCl_2_·6H_2_O, 1.2 NaH_2_PO_4_, 20 NaHCO_3_, 2.5 CaCl_2_·2H_2_O and 11 glucose) containing 500 μM probenecid (Sigma-Aldrich) to prevent dye leakage, was used.

The preparations were then placed in a recording chamber, superfused with 37°C Krebs solution (perfusion rate 8.5 ml/min) and mounted on an inverted epifluorescence microscope (Zeiss Axio Observer A1, Carl Zeiss, Jena, Germany) equipped with a high speed monochrome camera (Zeiss AxioCam HSm) and software (Zeiss Axio Vision 4.8) for acquisition and analysis. Fluo-4 AM was excited using a blue light emitting diode (LED) Luxeon III (3 W, 470 nm dominant wavelength, Philips Lumiled, Phillips, Hamburg, Germany) and the signals were detected with a filter cube F26-514 Bright Line FITC BP (excitation: HC475/35, dichroic: 499, emission: HC530/43, AHF Analysentechnik, Tübingen, Germany) using 20x objective (A-Plan, NA = 0.25, Zeiss) (Nemethova et al., 2013). The system measured relative changes in fluorescence (ΔF/F) of Fluo-4 AM monitoring changes in [Ca^2+^]_i_.

### Stimulation of MCs and neurons

Depending on the stimulus, [Ca^+2^]_i_ transients were recorded for 45 s up to 370 s using a frame rate of 0.5 or 1 Hz. Under basal condition the variation in background fluorescent was ±1%ΔF/F. A three-fold standard deviation (3%) was defined as threshold for genuine cell activation. Basal, non-stimulated [Ca^+2^]_i_ transients were recorded for 45 s and 0.5 Hz.

We selectively stimulated the MCs by IgE receptor cross linking using a monoclonal antibody (mAb) 22E7 to the high-affinity IgE receptor (Fc epsilon RI) (Riske et al., 1991). MAb 22E7 was generously supplied by Genentech-Roche, San Francisco, US. A 1 ml syringe was filled with 10 μg/ml of the antibody in order to locally perfuse an area of 0.05 mm^2^ (corresponding to 66% of the total field of view using the x20 objective) containing one submucous ganglion and mast cells located nearby. According to our previous studies using spritz pulse application, a 1:10 dilution of the applied substance has to be considered for the local perfusion as well (Breunig et al., 2007). The local perfusion via a micro perfusion pump (Micro4, WPI Sarasota FL, US) lasted 60 s at a speed of 0.1 μl/s.

To confirm that mAb22E7 is able to cause MC degranulation we incubated human submucous plexus preparations (size of 5 mm^2^) either with mAb22E7 (10 μg/ml) for 60 s or in Krebs buffer alone (control). After 60 s the supernatants were immediately removed and filtered (0.2 μm). Supernatants and tissues were stored at −80°C. We quantified the histamine concentrations in both the supernatants and the tissues using liquid chromatography tandem-mass spectrometry (targeted LC-MS/MS). Ten microliters of the supernatants were mixed with 10 μl of 1 μM Histamine-d4 (internal standard, Santa Cruz Biotechnology, Heidelberg, Germany), subsequently dried in a SpeedVac vacuum concentrator. The tissue samples were first dissolved in 30% methanol, homogenized, sonicated for 10 min and centrifuged (13,000 g for 10 min). Four hundred and sixty microliters of the liquid extract was then mixed with 10 μl of 1 μM Histamine-d4 and dried. Pre-column derivatisation of all samples was done using phenylisothiocyanate (PITC). Briefly, a 200 μL mixture consisting of 5% PITC dissolved in pyridine, ethanol, and 0.1% NH_3_ in a 1/1/1 ratio was added to the dried samples. Samples were shaken at 25°C for 20 min and were subsequently dried and then resuspended in methanol containing 5 mM ammonium acetate and finally diluted with water to reach a methanol/water ratio of 70:30. The analysis was performed on a triple quadrupole QTRAP 5500 LC-MS/MS system operating in positive ESI mode (AB Sciex, Framingham, MA, USA) equipped with a 1,200 series binary pump (Agilent, Santa Clara, CA, USA) and coupled to an HTC pal autosampler (CTC Analytics, Zwingen, Switzerland). Chromatographic separation was achieved using a Zorbax Eclipse XDB-C18 column (length 150 mm, internal diameter 3.0 mm, particle size 3.5 μm; Agilent, Waldbronn, Germany). Eluent A consisted of 0.2% formic acid in water. Eluent B consisted of 0.2% formic acid in acetonitrile. Histamine was measured in scheduled multiple reaction monitoring (sMRM). For quantification, an 9-point calibration was performed (0–50 μM) using pure histamine. Data analysis was done using Analyst 1.5.1® software (AB Sciex). All results were normalized to the weight of the tissue.

We specifically stimulated the neurons in the human submucous plexus by transmural electrical field stimulation (EFS). The EFS of the nerves was achieved by two platinum electrodes and a constant voltage stimulator (Typ 215/I, Hugo Sachs Elektronik, Hayard-Apparatus GmbH, March-Hugstetten, Germany). The stimulus parameters were: 10 s 50 Hz pulse train with 0.5 ms pulse duration at 25 V. The rather high stimulus frequency was based on early studies demonstrating maximal effects on muscle tone at a frequency of 50 Hz (Paton and Vane, 1963). The response to EFS did not run down. We calculated the average MC response index (peak [Ca^+2^]_i_ as %ΔF/F x % responding MCs) of tissues which had been stimulated with transmural EFS for at least three to eight times (6 tissues, 6 ganglia, 23 MCs) and compared the values with responses to the first EFS (19 tissues, 19 ganglia, 115 MCs). The median MC response index did not vary between these two groups [first EFS: 235 (170/546) vs. third-eighth EFS: 560 (146/1531)] (*P* = 0.546, Mann–Whitney Rank Sum Test).

For pharmacological studies the following substances were added to the perfusing Krebs solution: 1 μM tetrodoxin (TTX, Alexis, Lausen, Switzerland) perfused for 20 min to block nerve conduction; 10 μM BIBN-4096 for 60 min (Synonym BIBN 4096BS, Tocris Bioscience Bristol, UK) dissolved in dimethyl sulphoxide to block calcitonin gene-related peptide (CGRP) receptor 1; a mix of neurokinin (NK) receptor antagonists (NK1: SR140333B; NK2: SR48968C; NK3: SR142801, all of them 1 μM) for 20 min (all Sanofi-Aventis, Paris, France) dissolved in dimethyl sulphoxide to block effects of substance P; and the vasoactive intestinal peptide receptor (VPAC) antagonist PG97-269 (25 μM) for 20 min (Phoenix Pharmaceuticals, Burlingame, CA, USA) dissolved in deionized water to block effects of the vasoactive intestinal peptide (VIP). Additionally, nicotine (100 μm; Sigma) was applied in some experiments via pressure pulse application for 600 ms (Buhner et al., 2009).

### Immunhistochemistry

As described previously, outline of ganglia and neurons could be detected during the rise of intracellular [Ca^+2^] accompanied by the increase in Fluo-4AM fluorescence (Michel et al., 2011). Neurons were identified and distinguished from glia cells by their typical appearance (neurons are larger and round shaped, glia are much smaller and typically oval) and by their response to nicotine or electrical stimulation (only neurons respond directly; Michel et al., 2011; Ostertag et al., 2015). A vital staining of MCs immediately after the recordings was achieved by a 60 min incubation of the tissue with a R-phycoerythrin-conjugated mouse anti CD117 (c-kit) antibody (1:200; 104D2 directed against an extracellular epitope, EXBio, Praha, Czech Republic). After staining, the tissue was washed with Krebs buffer for 5 min. Images of the labeled MCs were acquired using a 3W green LED (LE TA2A true green (521 nm) 700 mA (OSRAM GmbH, Munich, Germany) and a filter cube (F46-004 ET filter set, excitation ET 545/25, dichroic 565, emission ET 605/70; AHT Analysentechnik, Tübingen, Germany). The overlay of the Fluo-4AM and CD117/C-Kit images allowed analyzing responses of individual MCs in the field of view.

CD117/C-Kit positive MCs were clearly distinguishable from interstitial cells of Cajal (ICC) based on their round shape compared to the star- or radial shaped ICC morphology. In some tissues we verified the MC identification using the mouse anti-MC tryptase (1:2,000; Chemicon GmbH, Limburg Germany). In all cases we were able to show that MCs were co-labeled by the c-kit and the MC tryptase antibodies. Enteric neurons were selectively labeled using sheep anti PGP 9.5 (1:20,000; The binding site, Birmingham, UK). Secondary antibodies coupled with Cy 3 (Dianova, Hamburg, Germany) or CF640R (Biotium, Fremont, CA, US) were used. The fluorescence was detected by an epifluorescent microscope (BX61 WI, Olympus, Hamburg, Germany).

### Data analysis

As parameter for cell activation the maximum intracellular [Ca^2+^]–increase relative to resting light level (%ΔF/F) was determined for each application. Additionally, the time (s) from application to the peak [Ca^+2^]_i_ responses and the relative proportion of responding MCs in the field of view using a x20 objective (0.08 mm^2^) was determined. As parameter for the overall MC activation a MC response index as the product of the peak [Ca^+2^]_i_ response (%ΔF/F) and the percentage of responding MCs was calculated.

The maximal [Ca^+2^]_i_ increase and the percentage of responding neuronal area (taking the area responding to nicotine as 100%) per ganglion were multiplied to obtain the Ca-neuroindex. The application of nicotine was used to identify and distinguish neurons from glia cells, and the nicotine responsive cells matched PGP 9.5 immunoreactive neurons (Mueller et al., 2011; Ostertag et al., 2015). Based on staining with an antibody recognizing α1, α3, and α5 subunits 98% of human submucous neurons express nicotinic receptors (own unpublished results). Nevertheless, we cannot claim that the nicotine responsive neurons represent all neurons in a given ganglion. In our hands, nicotine is the most reliable tool to selectively activate enteric neurons.

We measured the distances between responding MCs and nerve fibers or responding ganglia using the overlay of the Fluo-4AM and CD117/C-Kit images and the x20 objective. In experiments with EFS induced nerve stimulation we measured the minimum distance between the activated neural structures (nerve fibers or border of responding ganglia) and the center of the responding MCs. This was possible because we could identify all sufficiently dye loaded vital neural structures in the field of view by EFS. This was not possible in the experiments with anti-IgE induced MC stimulation. Here we did not use EFS stimulation to avoid premature mast cell activation and mediator release. We believe that the one distance measured is representative for nerve-MC as well as MC-nerve signaling. Numbers of tissue, ganglia, and MCs are given in sequence without further specification, e.g., a result based on experiments from 4 tissues, 5 ganglia, and 40 MCs is presented as (4/5/40). Data are expressed as the median with the 25th and 75th quartiles given in parenthesis and separated by slash. Statistical tests and graphs were performed using SigmaPlot 12.5 (Systat Software Inc., Erkrath, Germany). All experiments with blockers were done in a paired fashion. Normally distributed data were analyzed by paired *t*-test; not normally distributed data were analyzed by the Wilcoxon signed rank test, the Mann–Whitney Rank Sum Test and the Friedman repeated measures analysis of variance with *post-hoc* all pairwise multiple comparison by Tukey test. A Bonferroni procedure was applied. Fisher's exact test was used to test whether MC [Ca^2+^]_i_ oscillations were linked to disease, localization of surgical specimens or gender. For all tests a *P* < 0.05 was considered significant.

## Results

### Basal [Ca^2+^]_i_ transients in MC

**Figure 1:**
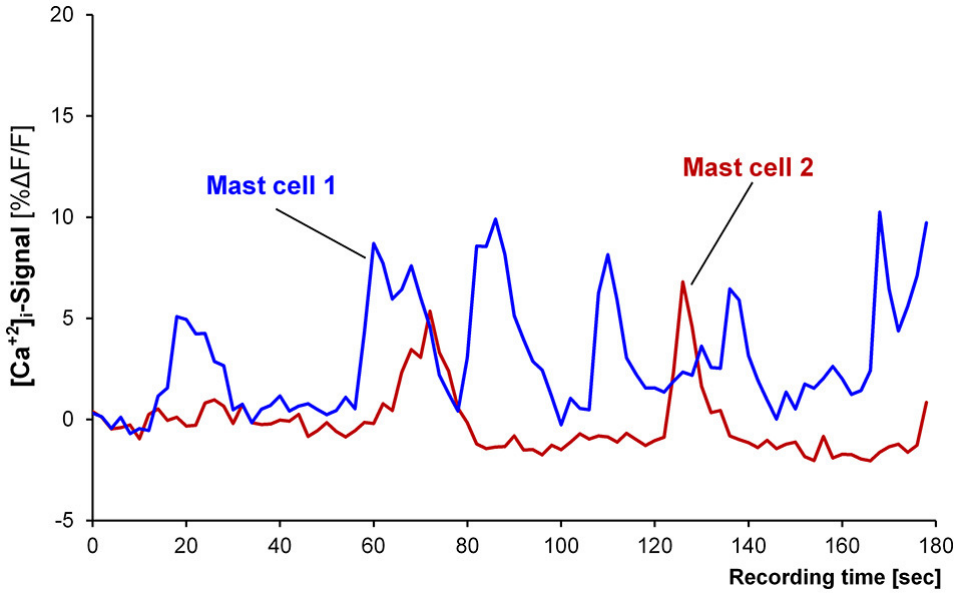
Recording from two spontaneously active mast cells. Mast cell 1 (blue trace) is showing six [Ca^2+^]_i_ peaks and mast cell 2 (red trace) is showing two peaks.

Basal activity was recorded and analyzed from 165 MCs in 22 tissues. Twenty-eight MCs (17% of all MCs) in 13 tissues showed spontaneous oscillations in [Ca^2+^]_i_ with an average frequency of 0.5 per min (0.3/0.7; Figure 1). Their median amplitude was 8.1% ΔF/F (5/11), their median duration about 16 s (7/25). Spontaneous neuronal oscillations in [Ca^2+^]_i_ were not observed in any of the 22 tissues (23 ganglia). We analyzed 13 tissues with and 9 tissues without oscillating [Ca^2+^]_i_ transients in MCs. There was no association with age (67 ± 11 vs. 58 ± 13; *P* = 0.053, *t*-test), gender (female/male: 4/9 vs. 1/8; *P* = 0.360), intestinal region (small intestine/large intestine: 6/7 vs. 4/5; *P* = 1.0), or the underlying disease (carcinoma/non-carcinoma: 13/0 vs. 8/1; *P* = 0.409). For further experiments only those MCs were included, which did not show spontaneous basal activity.

### MC to nerve signaling

**Figure 2:**
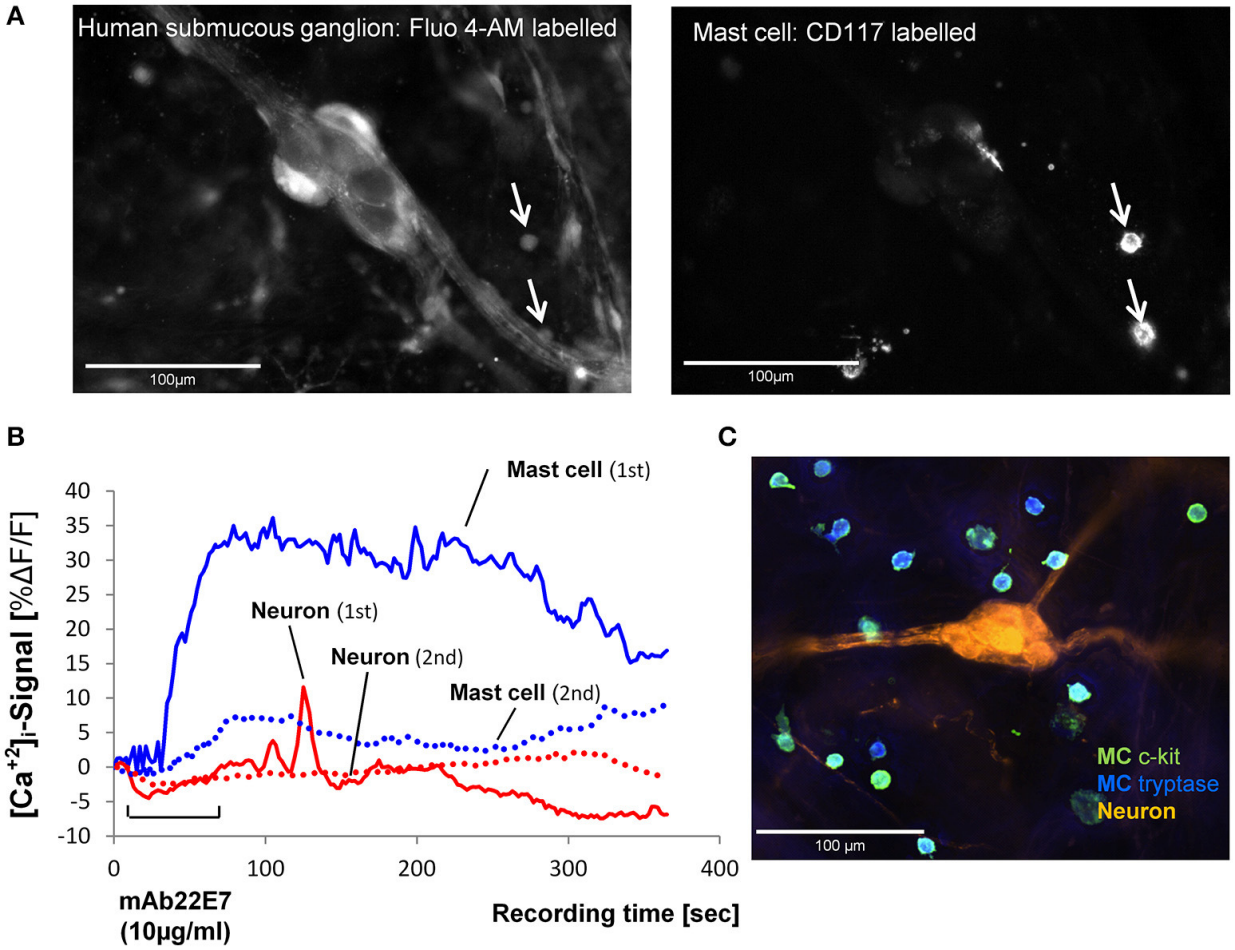
**(A)** Image on the left panel shows a human submucous ganglion stained with the Ca^2+^ sensitive dye Fluo-4AM. The arrows point toward two MCs which were identified by R-phycoerythrin-conjugated mouse anti CD117 (c-kit) labeling shown in the right panel. **(B)**. Traces of [Ca^2+^]_i_ transients in a mast cell (blue trace) and a submucous neuronal cell body (red trace) in response to mast cell activation by FcεRI receptor crosslinking by mAb22E7 micro-pefusion (10 μg/ml for 60 s). The mast cell showed a strong, long-lasting increase of [Ca^2+^]_i_ (1st mAb22E7). This response was strongly reduced after a second mAb22E7 application (see dotted line). Also, the response of the submucous neuron (solid line shows the signal after the first mAb22E7 application) is greatly reduced after the mAb22E7 application of (see dotted line). Panel **(C)** demonstrate a 100% overlap between anti-MC tryptase (blue) anti CD117 (green) MC staining. Enteric neurons were labeled with anti-PGP 9.5 (orange).

We selectively stimulated the MCs in close proximity to submucous ganglia by IgE receptor crosslinking using the monoclonal antibody mAb22E7 (10 μg/ml). The mAb22E7 had no direct effect on neurons. This conclusion was based on the finding that none of the 15 neurons (3 tissues and 3 ganglia) which responded to a 1 s pressure pulse application of nicotine with a Ca-neuroindex of 1186 (891/1448) responded to mAb22E7 application directly onto the ganglia [Ca-neuroindex 0 (0/0)]. Additionally, the mAb22E7 application had no effect on sensitivity of the neurons as a second application of nicotine 20 min later evoked a comparable Ca-neuroindex of 1973 (871/3320). This agreed with our previous study which demonstrated that mAb22E7 did not evoke spike discharge in human submucous neurons (Schemann et al., 2005). In contrast, 63% (43/75) of all MCs in the field of view responded to mAb22E7 application with a strong and long-lasting [Ca^2+^]_i_ increase (Figure 2; Table 1). In five tissues and 34 MCs, mAb22E7 was re-applied 20 min later. The MC activation was strongly reduced (Figure 2) from 20.4% ΔF/F (12.5/35.9) to 6.1% ΔF/F (0.0/11.9; *P* = 0.002). Likewise, the MC response index dropped from 1576 (821/2521) to 366 (88/735; *P* = 0.01). [Ca^2+^]_i_ transients in submucous neurons subsequently to MC activation with mAb22E7 were observed in 14 out of 35 ganglia, however only in a limited number of neurons (Figure 2). The calculated median percentage of responding neuronal area showing activation (taking nicotine-responsive neurons as 100%) was therefore 0% (0/5.9; Table 1). The maximum of the neuronal [Ca^2+^]_i_ signal was 6.6%ΔF/F. It occurred 208 s after the onset of the stimulus and thus clearly later than the maximum MC responses. However, since the neuronal responses were recorded so seldom we decided to forgo further pharmacological characterization of this phenomenon.

**Table 1:** Mast cell and neuronal responses to mast cell activation by IgE receptor cross linking with mAb22E7.

	**Mast Cells^a^**	**Nerve cell bodies**
Peak amplitude [Ca^+2^]_i_ %ΔF/F	19.5 (11.2/38.8)	6.6 (5.1/11.6)
Time of peak [Ca^+2^]_i_ [s]	90.0 (63.5/144.0)	208.0 (83.8/258.8)
Responding cells [%]	63 (43/75)	0 (0/5.9)
Response Index [%ΔF/F x % responding cells]	1,542 (800/2,429)	0 (0/36)

a *All values are medians with 25 and 75% quartiles in parenthesis and based on 29 tissues, 35 ganglia, and 223 mast cells*.

To confirm that mAb22E7 is able to cause MC degranulation in our human submucous plexus preparations we incubated the tissues (*n* = 2) with either mAb22E7 (10 μg/ml) or with Krebs buffer (control) for 60 s and quantified the histamine concentrations in both the supernatants and the tissues. After control incubation the histamine concentration in the supernatants was on average 0.19 ng/ml^*^mg. The incubation with mAb22E7 increased this value to 4.13 ng/ml^*^mg. The tissue histamine concentrations were higher, however, they followed a similar patterns (control: 105.1 ng/ml^*^mg vs. 224.4 ng/ml^*^mg after mAb22E7 incubation).

### Nerve to MC signaling

**Figure 3:**
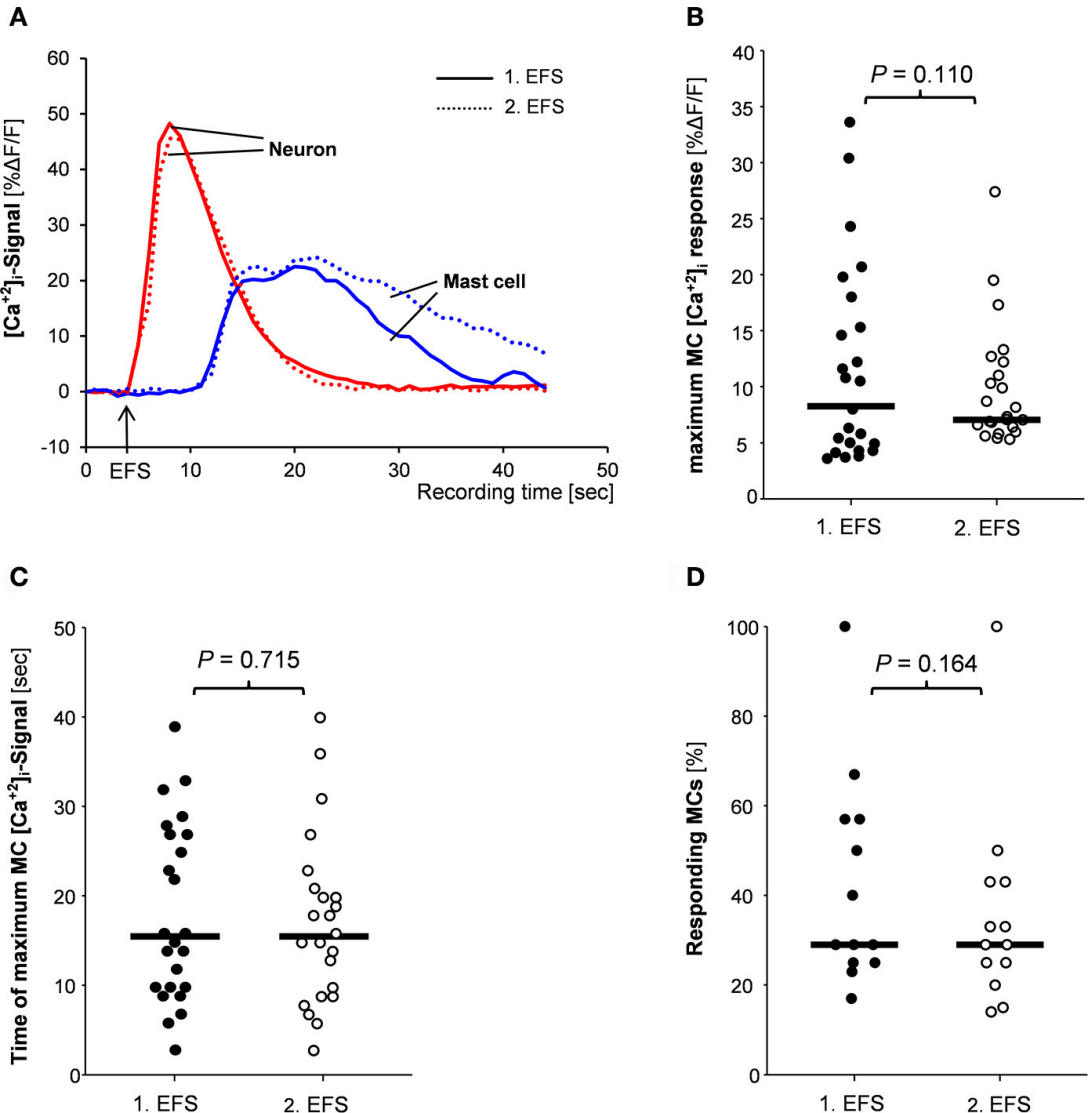
**(A)** Traces of [Ca^2+^]_i_ transients of a neuron (red trace) and a mast cell (blue trace) in response to nerve stimulation (EFS). Neuronal responses occurred instantaneously with the stimulus, while MC activation occurred after a time delay. Both, neuronal responses and mast cell activation were reproducible (compare solid and dotted traces). There were no differences between 1st and 2nd EFS evoked MC signaling regarding amplitude of [Ca^2+^]_i_ peaks **(B)**, time point of the [Ca^2+^]_i_ peak **(C)** or percentage of responding mast cells **(D)**. *P*-values based on Wilcoxon Signed Rank Test. Results based on 13 tissues, 13 ganglia, and 78 mast cells.

We selectively stimulated the neurons via EFS which evoked responses instantaneously upon the onset of the stimulus and reached the maximum of 38.4%ΔF/F (27.1/51.7) 2 s (2/3) after the onset of the stimulus (34 tissues/35 ganglia; Figure 3A). In these tissues a total number of 168 MCs were identified by IHC staining. Thirty-three percent (22/66) of them showed a [Ca^2+^]_i_ signal following the electrical nerve stimulation. The [Ca^2+^]_i_ peak of 8.0%ΔF/F (4.3/14.8) was reached later than the neuronal peak maximum, i.e., 11 s (7/25) after the onset stimulus. The MC response lasted 19 s (15/31; Figure 3A). The minimum distances between the EFS activated nerve fibers or ganglia and the responding MCs were 5 μm (1/12) or 32 (11/53), respectively.

EFS evoked nerve and MC responses were reproducible (Figure 3A). In 57% of the tissues MCs showed similar [Ca^2+^]_i_ transients after two EFS 20 min apart. Parameters for the MC responses were exemplarily calculated for 78 MCs in 13 tissues (Figures 3B–D). Neither the peak amplitude nor the time point of the maximum [Ca^2+^]_i_ signal or the percentage of responding MCs differed significantly between two EFS. For further pharmacological studies, only tissues with reproducible MC activation were used.

**Figure 4:**
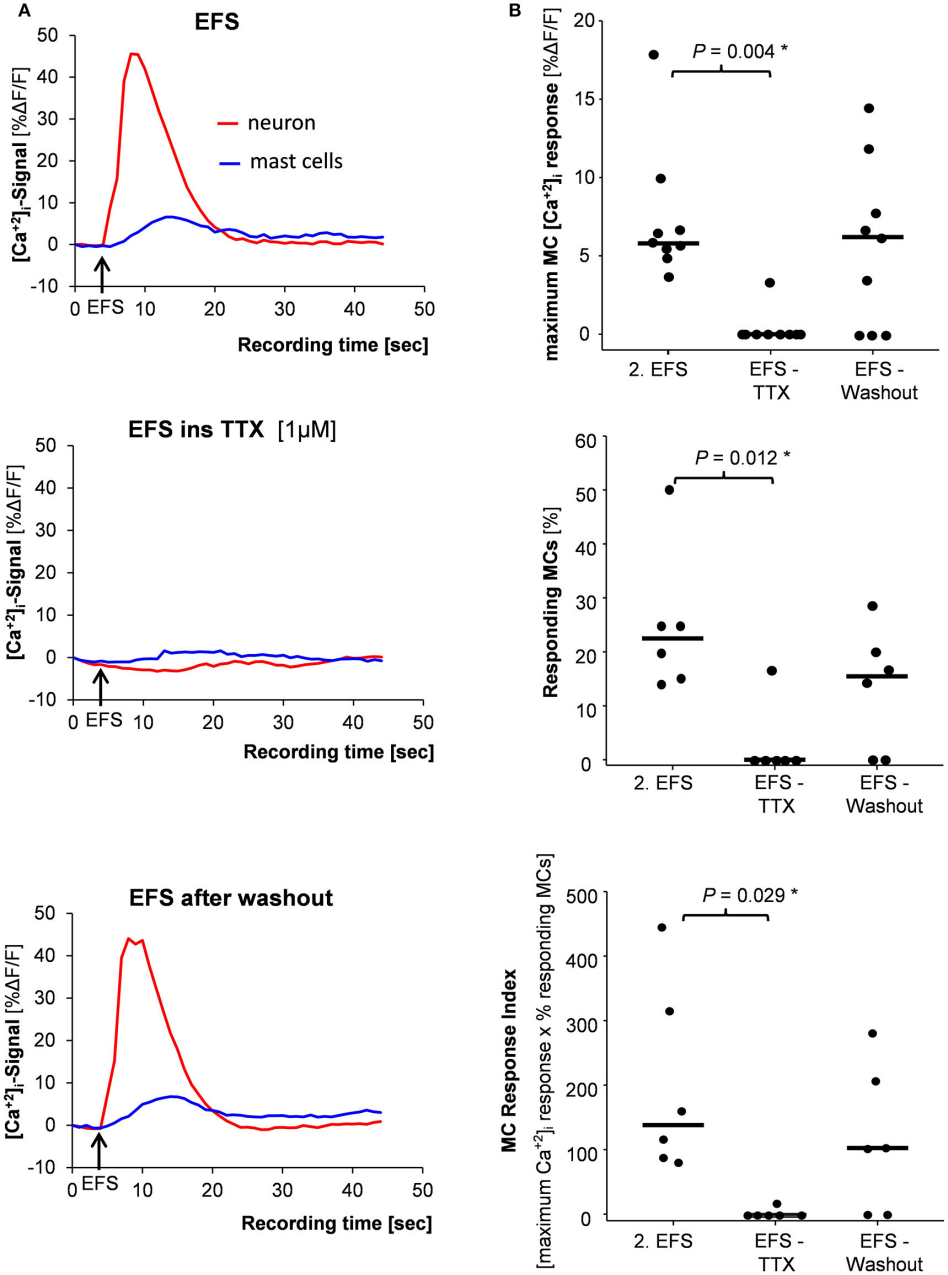
**(A)** Mast cell activation (blue trace) evoked by nerve stimulation (EFS) (red trace) was completely blocked by the nerve blocker tetrododoxin (TTX) (middle panel) and returned after TTX washout (lower panel). **(B)** Correspondingly, the amplitude of the [Ca^2+^]_i_ signal (upper panel), the percentage of responding mast cells (middle panel) and the mast cell response index (lower panel) were significantly reduced by TTX. *P*-values based on Friedman analysis of variance on ranks and *post-hoc* Tukey test; results based on 6 tissues, 6 ganglia, and 39 mast cells.

TTX (1 μM) blocked the EFS evoked nerve as well as the MC activation which both recovered after 60 min washout period (Figures 4A,B). Importantly, TTX had no direct effect on MC activation by mAb22E7. After 20 min perfusion with TTX, mAb22E7 application activated 88 % of the MCs in the field of view with a median maximum [Ca^2+^]_i_ signal of 33.7% ΔF/F (23.9/44.1; 2/2/25). These values were comparable to mAb22E7 effects without TTX (see Table 1).

**Figure 5:**
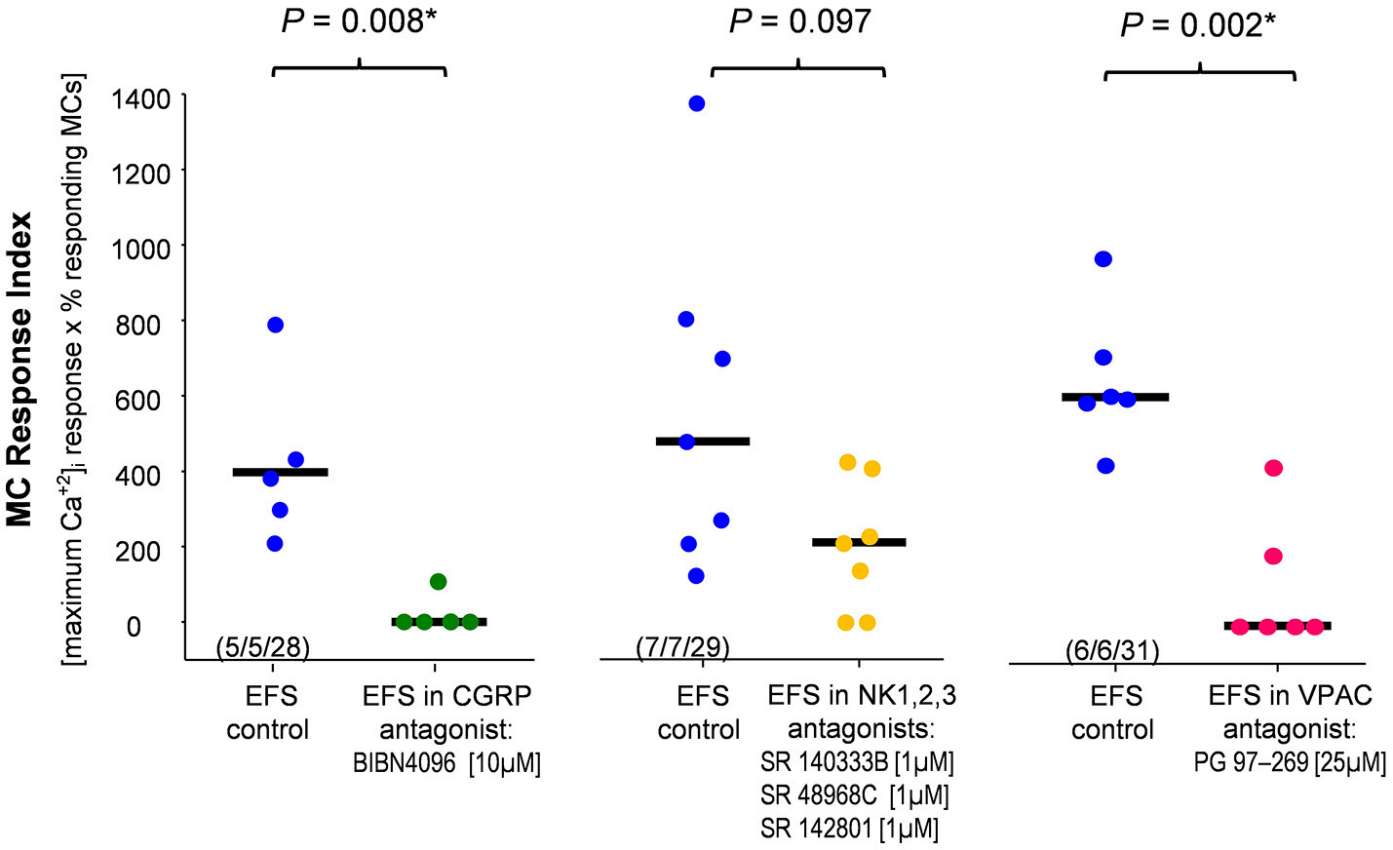
Calcitonin gene-related peptide (CGRP), vasoactive intestinal peptide (VIP) but not substance P contributed to nerve evoked mast cell activation. The CGRP antagonist and the VIP antagonist significantly reduced the EFS evoked mast cell response index. Co-application of neurokinin receptor antagonists had no significant effect. *P*-values based on Mann–Whitney Rank Sum Test; (tissues, ganglia, mast cells).

Next, we investigated which neurotransmitters were involved in the nerve-MC signaling. We focused on the most likely candidates CGRP, SP and VIP (see section Introduction). We repeated the EFS in the presence of the CGRP receptor antagonist BIBN-4096 (10 μM; Doods et al., 2000) and analyzed MC activation by calculating the MC response index. BIBN-4096 strongly reduced the MC responses (Figure 5). In contrast, blocking SP effects using a mixture of NK1, NK2, and NK3 receptor antagonists (all 1 μM) did not significantly reduce the MC activation (Figure 5). Furthermore, the VPAC receptor antagonist (25 μM, 20 min) significantly reduced the EFS evoked MC activation (Figure 5). In summary, the results of the pharmacological experiments indicate that CGRP and VIP rather than NK receptor meditated SP played a role in nerve mediated MC activation in human submucous plexus.

## Discussion

This is the first study to demonstrate nerve-MC signaling at a cellular level in intact intestinal tissue. We used [Ca^+2^]_i_ imaging in human submucous plexus to synchronously record MC and nerve activity after selective stimulation of nerves and mast cells. We presented strong evidence that MCs were activated by nerves based on the finding that the nerve blocker TTX abolished [Ca^+2^]_i_ signals in neurons and MCs. We ruled out TTX effects on mast cells because mAb22E7 induced activation of MCs was not TTX sensitive. This agreed with studies showing the lack of functional expression of voltage and TTX sensitive sodium channels in MCs (Bradding et al., 2003). The nerve-MC signaling involved the neuropeptides CGRP and VIP rather than SP acting through NK receptors. Nerve activation upon specific stimulation of MCs via IgE receptor cross-linking was also recorded, however to a much lesser extent. All in all, results suggested nerve to MC as well as MC to nerve communication.

Compound 48/80 was widely used to promote MC degranulation and to study MC to nerve communication (Janiszewski et al., 1994; De Jonge et al., 2004; Rychter et al., 2011; Wang et al., 2014; Bell et al., 2015). In a previous study, however, we revealed a direct and strong excitatory action of compound 48/80 on enteric neurons and visceral afferents (Schemann et al., 2012). Therefore, functional changes measured in tissue or animal models may be confounded by neural effects of compound 48/80. Classical MC activation in tissues occurred through cross-linking of adjacent high affinity receptors for the Fcε portion of IgE (FcεRI) on the MC surface by a multivalent IgE-antigen. Receptor cross-linking was induced by the antibody mAb22E7 that has been previously used to activate human intestinal mast cells (Bischoff et al., 1999; Sellge et al., 2005). We used it at 10 μg/ml which corresponded to results that mAb22E7 evoked a maximum histamine release at 2.5–10 μg/ml (Riske et al., 1991). This suggested that we used sufficiently high mAb22E7 concentrations. We verified MC mediator release in our tissues by showing that histamine release increased by more than 20-fold during brief exposure to mAb22E7. Additionally, mAb22E7 was specific for MCs as activation of other cells, in particular neurons, was not observed in our human submucous preparations.

In the present study around 63–88% of all MCs in the field of view showed a strong and long lasting [Ca^+2^]_i_ increase after mAb22E7 stimulation. The failure to activate all MCs may be due to insufficient dye loading or non-viable MCs. In addition, to avoid spill-over to adjacent areas, we adjusted the microperfusion system such that not the entire field of view was exposed to the drug solution. The MC [Ca^+2^]_i_ response closely resembled the signaling pattern which has been related to MC degranulation (Rychter et al., 2011; Wang et al., 2014; Gaudenzio et al., 2016). Strikingly, neural activation in terms of [Ca^+2^]_i_ transients, was rarely observed and if present of small amplitude. The activation was clearly triggered as the neuronal signal always occurred after MC responses. Nonetheless, we could not provide experimental proof as the neural response after MC stimulation was too rare and moreover not reproducible.

The delay in [Ca^+2^]_i_ transients between MC and enteric neurons was around 100 s. There are a number of factors influencing the timing of responses between MCs and nerves. This includes delays between [Ca^+2^]_i_ peak and release of MC mediators, diffusion of mediators to the neuronal target, activation of G-protein coupled receptors (GPCRs) and GPCR induced enhanced [Ca^+2^]_i_ signal. Single cell measurements in cultured mast cell lines found a 30–60 s delay between the [Ca^+2^]_i_ increase and secretion of mediators (Kim et al., 1997). The first spike after application of histamine onto human submucous neurons is discharged after 1–2 s (Breunig et al., 2007). The [Ca^+2^]_i_ peak in response to enhanced spike discharge occurred much later. This is the reason that we observed delays of about 20 s between application of PAR agonists (among them tryptase) and the peak [Ca^+2^]_i_ response in human submucous neurons (Mueller et al., 2011; Ostertag et al., 2017). There was a delay of several minutes between [Ca^+2^]_i_ increase and degranulation (Gaudenzio et al., 2016). Last but not least, there is an unknown time needed for diffusion of MC mediators to their receptors on enteric neurons. Therefore, our average delay of around 100 s are somehow expected and within the range suggested by the above referenced studies.

Previously, we demonstrated that a “mast cell mediator cocktail,” which was released from isolated human intestinal mast cells by IgE receptor cross linking (Schemann et al., 2005) as well as single MC degranulation products like histamine (Breunig et al., 2007) and proteases (Mueller et al., 2011) evoked strong and immediate action potential discharge in human and guinea pig submucous neurons. Thus, the question remained why the neuronal response after MC stimulation in the present study was observed so sporadically. One possible explanation is that mediator release from a limited number of nerve-associated MCs may be not sufficient to activate neurons nearby. Neuronal cell bodies were on average 32 μm away from the activated mast cell. Although, volume transmission may happen over even much larger distances (Zoli et al., 1999), another scenario is at least as plausible. The signaling of MCs to nerves likely occurred at the neurites and activation of the nerve cell bodies may be a consequence of an activation of such extra-ganglionic processes. Signaling between MCs and neurites may not necessarily activate a neuronal cell body in the ganglion-nearby MCs but in more distant ganglia outside our recording area. With the limited amount of mAb22E7 it was not possible to perfuse the entire tissue to test this hypothesis. Based on the following finding, the above discussed methodological limitations cannot fully explain the rare occurrence of MC to nerve signaling. Although, the transmural stimulation of all nerves evoked a strong MC activation we never observed a late onset [Ca^+2^]_i_ signal in nerves. This would be expected if release of MC mediators feedback on nerve activity. Weighing all the above discussed arguments, we conclude that an MC to nerve signaling is a rather rare event in our samples of macroscopically normal tissue. Having said that, a MC to nerve signaling may be more prominent in disease states like IBS or IBD where the number and/or the reactivity of MCs in close proximity to nerves is increased (Raithel et al., 2001; Barbara et al., 2004, 2007; Cenac et al., 2007; Guilarte et al., 2007).

Nerve to MC signaling was frequently observed and occurred in 33% of MCs. The transmural EFS used in the present study was stimulating the entire preparation and thus activated all submucous as well as extrinsic nerves. In the present study we calculated a median distance of 5 μm between activated nerve fibers and MCs. The spatial resolution of the imaging set up did not allow in tissue recordings from single axons to follow the spread of action potentials from the cell body along the axon to the varicose endings at the transmission to MCs. The present study provided direct evidence that nerves activated MCs in intestinal preparations. This conclusion was supported by the finding that the [Ca^2+^]_i_ signals in MCs were completely blocked by TTX. The mediators involved were CGRP and VIP rather than SP. In human colonic submucous plexus CGRP is exclusively expressed by extrinsic, very likely sensory, nerve fibers with intraganglionic terminals as well as varicose endings outside ganglia (Schneider et al., 2001). MCs in the human intestine express CGRP receptors (Wang et al., 2014). A major result of the present study was that blocking these receptors with the selective and high-affinity antagonist BIBN-4096, strongly reduces the MC activation upon nerve stimulation. This clearly stressed the role of CGRP in nerve mediated activation of MCs in intact human submucous plexus. These findings are in accordance with the results in cell cultures (De Jonge et al., 2004; Rychter et al., 2011) and rodent plexus preparations (Wang et al., 2014). VIP was also involved in the nerve mediated MC activation. The VPAC receptor antagonist PG97-269 (Gourlet et al., 1997) reduced nerve evoked MC activation. According to binding studies, the affinity of PG97-269 is higher to VPAC1, however it binds and inhibits VPAC2 actions as well (Gourlet et al., 1997). VIP is a prominent neuropeptide, produced by neurons in human submucous plexus (Schneider et al., 2001). Functions are ranging from neurotransmission to immunomodulation (Said and Rosenberg, 1976), the latter was reflected by the ability of VIP to induce degranulation of rat brain MCs (Tunçel et al., 2005). The vast majority of VIP positive structures in human intestinal preparations originated in the enteric nervous system. VIP positive cell bodies have not been found in dorsal root ganglia of human fetus or newborn infants (Charnay et al., 1985). We therefore believe that an earlier study suggesting that VIP is a sensory transmitter rather described a capsaicin evoked release of CGRP which then activated VIP release from enteric neurons (Maggi et al., 1989). We have no final explanation for the finding that the CGRP as well as the VIP antagonist almost abolished the MC response to EFS. One reason, though speculative, may be that both act in such a synergistic way that blocking the action of one is also abolishing the response to the other mediator or inhibiting it to such an extent that we cannot resolve the response anymore. We have no evidence for any unspecific effects of the blockers and we applied the protocols and the concentrations also used by others (BIBN4096: (De Jonge et al., 2004; Rychter et al., 2011); VPAC antagonist: (Krueger et al., 2016).

Surprisingly, we found no evidence that SP was involved in MC activation. However, we have to acknowledge that the effects of the neurokinin receptor antagonists barely missed significance. It seems that some MCs responded nicely other not at all to blockade of SP action. This agrees with the finding that only up to 17% of mast cells in human intestine express NK1 receptors (Wang et al., 2014). Substance P is only present in a small proportion of human submucous neurons and is, unlike in rodent intestine, very rarely co-localized with extrinsic CGRP positive fibers (Ekblad et al., 1989; Schneider et al., 2001). There is abundant evidence in rodent models that SP activated MCs (Janiszewski et al., 1994; Wang et al., 2014; Gaudenzio et al., 2016) and induced degranulation (Suzuki et al., 2001; Kulka et al., 2008). However, the presence of tachykinin receptors on mammalian MCs remained controversial (Lecci et al., 2006). Bischoff et al. (2004) showed that cultured human intestinal MCs do not constitutively express NK receptors, however, when primed by stem cell factor or interleukin-4, they started to express NK1 receptors and responded to high concentrations of SP with degranulation. Thus, NK receptor mediated SP effects on MCs may vary with their precondition.

We observed in few cases oscillations in [Ca^+2^]_i_ signals of MCs. Resting [Ca^+2^]_i_ fluctuation commonly occurred in MCs and was suggested to represent fluctuations in the filling state of internal Ca^2+^ stores (Millard et al., 1989; Narenjkar et al., 1999).

This is the first study to demonstrate nerve-MC interaction on a cellular level in intact human submucous plexus imaging [Ca^+2^]_i_ transients. According to these results we propose a functional nerve mast cell axis in the human gut. A nerve to mast cell signaling was more prominent than a mast cell to nerve signaling.

## Ethics statement

This study involved *in vitro* experiments on surgical waste tissue from human intestine. This study was carried out in accordance with the recommendations of the ethics committee of the Technical University of Munich with written informed consent from all subjects. All subjects gave written informed consent in accordance with the Declaration of Helsinki. The protocol was approved by the ethics committee of the Technical University of Munich (project approval 5242/11).

## Author contributions

SB: designed the study and performed the experiments as well as analysis and interpretation of data. She drafted the manuscript, approved the final version, and is accountable for all aspects of the work. NB: performed experiments as well as analysis and interpretation of data. She critically revised the manuscript. She is accountable for all aspects of the work. WG: performed experiments as well as analysis and interpretation of data. He critically revised the manuscript, approved it for publication and is accountable for all aspects of the work. PG: performed the histamine analysis. He critically revised the manuscript, approved it for publication and is accountable for all aspects of the work. ID, GC, and FZ: made the work possible by providing human samples and medical council. They critically revised the manuscript and approved it for publication. They are accountable for correct patient characterization and material handling. HD: She critically reviewed the manuscript for important intellectual content, approved it for publication and is accountable for all aspects of the work. MS: formed the concept and designed the study as well as made the work possible by obtaining funding. He critically reviewed the manuscript for important intellectual content, gave final approval for publication and is accountable for all aspects of the work.

### Conflict of interest statement

The authors declare that the research was conducted in the absence of any commercial or financial relationships that could be construed as a potential conflict of interest.

## Abbreviations

IBS: irritable bowel syndrome
IBD: inflammatory bowel diseases
ENS: enteric nervous system
[Ca^2+^]_i_: intracellular calcium-increase
TTX: tetrodoxin
CGRP: calcitonin gene-related peptide
VIP: vasoactive intestinal peptide
VPAC: vasoactive intestinal peptide receptor
SP: substance P
NK: neurokinin.
